# Developing a spectrum model of engagement in services for first episode psychosis: beyond attendance

**DOI:** 10.3389/fpsyt.2024.1429135

**Published:** 2024-10-03

**Authors:** M. Ferrari, K. MacDonald, J. Sabetti, T. Cowan, S. N. Iyer

**Affiliations:** ^1^ Douglas Research Centre, Douglas Mental Health University Institute, Montreal, QC, Canada; ^2^ Department of Psychiatry, McGill University, Montreal, QC, Canada

**Keywords:** engagement, disengagement, early intervention services, psychosis, grounded theory, recovery

## Abstract

**Background:**

Early intervention services (EIS) for psychosis have proven highly effective in treating first episode psychosis. Yet, retention or “engagement” in EIS remains highly variable. Dis/engagement as a contested concept and fluid process involving relationships between service providers and service users remains poorly understood. This study aimed to critically evaluate and explain the dynamic interplay of service provider-user relationships in effecting dis/engagement from an early intervention program for psychosis.

**Methods:**

Forty study participants, 16 service providers and 24 service users (19 current and 5 disengaged) from a Canadian EIS program, were administered semi-structured interviews. Qualitative analysis was conducted using grounded theory methods, with findings captured and reconceptualized in a novel explanatory model.

**Findings:**

A model of engagement with eight major domains of engagement in EIS positioned along a control-autonomy spectrum was developed from the findings, with Clinical engagement (attendance) and Life engagement (life activities) at opposite ends of the spectrum, interspersed by six intermediate domains: Medication/treatment, Symptoms/illness, Mental health, Physical health/wellness, Communication, and Relationships, each domain bearing uniquely on engagement.

**Conclusions:**

An examination of service user and service provider perspectives on the various domains identified in the spectrum model, and their dynamic interplay, reveals the complexity of choices faced by service users in engaging and not engaging with services.

## 1 Introduction

Psychotic disorders first occur in adolescence and early adulthood, shaping the lives of affected young people emotionally, socially and economically (1, 2), while also impacting family members and friends. Early intervention services (EIS) for psychosis demonstrate superior effectiveness to treatment as usual (3), yet rates of patient disengagement range from 1% to 41% (4). While signaling potential problems in service delivery, this variability also reflects the nature of engagement as a contested concept with widely varying definitions. A review (5) found that definitions and outcome variables varied broadly in studies on patient disengagement from EIS. For example, disengagement rates could include individuals who disengaged with or without clinician consent, transferred to another service, or moved away. Designated timeframes for disengagement, when used, tended to be arbitrary (e.g., three or six months without contact from last clinical appointment) or based on numbers of missed appointments. This heterogeneity in conceptualizations of dis/engagement and research outcomes prompted calls for a consensus definition of engagement (4–7), which can only be gained by acquiring a better understanding of dis/engagement processes in EIS for psychosis.

Clinical guidelines for EIS highly value service user engagement. The Australian EIS guidelines highlight the importance of patient-provider rapport, adding that performing clinical assessments should not take precedence over developing therapeutic relationships (8). Canadian provincial EIS guidelines (9) promoted individual and family engagement as a primary therapeutic goal; while the NICE guidelines emphasized engagement over risk management in clinical teams (10).

While little research has been done on factors of engagement, more is known about factors promoting disengagement. Poor or non-adherence to medication (11, 12), lack of vocational involvement (employment, education, training) (13, 14), cannabis use (13), duration of untreated psychosis, symptom severity at baseline, poor insight, substance abuse or dependence, lack of family involvement (6), and lack of transportation (15) have all been identified in quantitative literature as factors promoting disengagement from EIS. Additionally, treatment disengagement due to perceptions that mental health services do not address their understanding of illness or their needs, and fears of stigma are common among mental health service users (16). Disengagement rates have been reported as particularly high among individuals from minoritized racial/ethnic groups, partly attributable to a lack of cultural sensitivity in delivering care (17). Similarly, immigrants in a Canadian study had three times higher odds of disengagement from EIS than non-immigrants, with distrust and stigma identified by the authors as potential factors (18). Pathways to care have also been shown to strongly influence engagement and treatment outcomes among service users (19–21). For instance, when access to treatment followed a single help-seeking episode, the duration of untreated psychosis was shorter and length of engagement longer; whereas hospitalization tended to delay access to treatment and result in treatment dropout (22). By contrast, strong patient-therapist alliance (23) and youth-friendly service environments (24, 25) facilitated engagement. Two studies underlined the positive influence of therapy on engagement (26), particularly CBT (27).

Emerging research problematizes the notion that dis/engagement is uniquely related to decision-making processes among service users and considers the roles of other stakeholders, opening the possibility of a more complex explanatory model (28). One study described engagement in EIS and related challenges as an evolving process where service users negotiate and renegotiate competing priorities as an exercise in personal agency. Such exercises in negotiation demonstrated both the person’s ability to exercise choice and ways in which service structures or providers supported or constrained this process (28). Similarly, a genuine therapeutic patient-provider alliance, the dynamic ability to work together in the interest of problem-solving, based on goals, tasks and bond (29), is widely viewed as a vehicle for engagement (7, 21, 30). One meta-synthesis identified the quality of a therapeutic relationship with at least one EIS staff as the single most important factor in whether service users would have a positive or negative experience of engagement (31). A qualitative study suggested that service user disengagement may relate to unresolved decision-making around treatment due to lack of support, opposition, outside pressures or personal reasons (e.g., motivation) (32). A longitudinal study identified three processes leading to disengagement: a mismatch between service model and the individual, lack of shared purpose with the provider, and unforeseen individual circumstances (33). By contrast, in settings characterized by trusting relationships, dialogue, collaboration, and mutual understanding, all parties (service users, providers, and caregivers) felt that their priorities and needs were being met (21, 26, 27, 30).

As a clear and harmonized definition of dis/engagement in EIS for psychosis is not yet available (4–7), which presents difficulties and challenges for conducting research on this process and particularly for creating clinical guidelines, the need to comprehend dis/engagement processes in EIS for psychosis is essential. The present study seeks to expand previous research with an in-depth exploration of the engagement process as an ongoing interaction between service users following their individual paths to mental health recovery and wellness, and service providers working with them in a partnership-based relationship (29, 31, 34). The overall objective is to critically evaluate the dynamic interplay of perspectives between service users and providers as the key players in effecting dis/engagement in EIS for psychosis, capturing and reconceptualizing their views in a novel explanatory model.

## 2 Materials and methods

### 2.1 Study design and methodology

This qualitative study used grounded theory to inform data collection and generate the analysis. Grounded theory locates emerging theory in data gathered from different participants and/or using different methods of data collection. The postmodern iteration of grounded theory developed by Charmaz (35–37) and selected to guide this study avoids a rigid prescription of tasks in favor of a set of principles for theorizing peoples’ experiences in a novel construction of reality. Using an inductive process to develop theory from patterns found in empirical data, grounded theory is essential for re/theorizing engagement in EIS services and for guiding decisions about which issues should be examined in constructing various dimensions of the engagement construct. We also consulted the COREQ guidelines (38) to ensure completeness of the analysis (see **Supplementary Data Sheet 1**).

### 2.2 Setting

The study was conducted at a Canadian EIS program located in an outpatient facility serving individuals aged 14-35. Like other EIS in Canada, this program within the public health care system provides case management, family psychoeducation and support, medication management, and psychosocial interventions (e.g., CBT), without charge. These services are offered as a “menu” such that (aside from case management) service users can choose to use some services but not all, and to use different services at different times. This flexibility is designed to maximize continued contact with the service as a whole by allowing disengagement from specific services while providing all those that should be necessary, so that service users will not need to disengage from the service entirely. Service users are typically provided two years of service, after which they are discharged through a warm hand-off process to the most appropriate available community care for that specific individual. Regarding study eligibility, service users had to have a psychotic disorder not substance-induced or attributable to an organic brain disorder (e.g., epilepsy), have taken antipsychotic medication for 30 days or less, and not have an intellectual disability (measured as 70 or higher on a standardized intelligence test). Comorbid substance use diagnoses were not reasons for exclusion.

### 2.3 Sampling and recruitment

The study used conventional, purposeful, and theoretical sampling, following the Charmaz approach (2006). Initial participant sampling was conventional, with service user recruitment based on their experience in EIS, while recruitment of service providers was more purposeful, based on their assumptions and understanding of engagement. Theoretical sampling further aimed to include service users and providers with diverse standpoints, for example, service users with various relationships to the EIS program (active service users versus those who left the service) and providers with various training experiences and levels of seniority in EIS. In all, 40 study participants were recruited, 24 service users, 19 of whom maintained at the time of their interview some level of contact with the treatment team and five who had left treatment before completing the recommended 2-year course, and 16 service providers. **Table 1** presents participant and provider characteristics.

**Table 1:** Characteristics of study participants.

Service user characteristics (n=24)	N
Age	
Mean: 22.67; range: 17-34 years	
Gender	
Cis men	16
Cis women	6
Transgender ^1^	2
Ethnicity	
White	9
Person of color	13
No answer	2
Country of birth	
Canada	17
Other	6
No answer	1
Highest level of education	
Less than high school	4
High school	11
Post-high school; vocational degree	7
University; bachelor’s degree	1
No answer	2
Occupation	
Full-time/part-time school	12
Full-time/part-time work	5
No current occupation	6
No answer	2
Service provider characteristics (n=16)	N
Role in EIS	
Non-physician mental health professional (case manager, screening clinician)	11
Psychiatrist	5
Age groups	
21-30	2
31-40	7
41-50	3
51-60	4
Years of experience in EIS	
0-5	3
5-10	7
10+	6

^1^In an effort to preserve anonymity, we aggregated all transgender individuals into a single category.

### 2.4 Data collection and analysis

The authors developed semi-structured interview guides for purposes of the study, with feedback from service users and providers. The primary question for both participant groups concerned the meaning of dis/engagement in EIS services and, for service users, how this had changed over time. Both were asked about user involvement in treatment, service user-provider relationships, the role of medications, the meaning of wellness and, for service users, areas of their lives that were going well, or less well. The interview guides were used flexibly and had a conversational feel. Individual interviews lasting 40-90 minutes were conducted at a single session in a private room at a hospital by a skilled research assistant with no one else present. Interviewers kept field notes of the interviews.

Charmaz’s constructivist grounded theory approach underscores the iterative and inductive nature of data analysis, enabling the construction of theories firmly grounded in the data (36). Indeed, coding plays a critical role in identifying, organizing, and constructing new theories based on the collected data. The 40 interviews were audio recorded, transcribed verbatim and checked for accuracy. Atlas.ti (Version 1.6.0) was used to organize and manage the data. MF, who is familiar with grounded theory analysis, took the lead in performing the initial coding, open coding, and axial coding to support theory development; three other team members (KM, JS, TC) were involved in coding at different stages.

Following the grounded theory approach, data analysis began with a complex coding process, “the first step in moving beyond concrete statements in the data to making analytic interpretations” (36: p.43). Short code names were applied to each data segment, which was defined descriptively. At the second level, focused coding was used to establish constant comparisons and linkages between the identified codes and concepts. Axial coding is a fundamental component of grounded theory, which serves primarily to establish connections between a category and its subcategories, enabling a deeper understanding of the relationships within the data. Axial coding was used to define concepts/categories, such as the eight domains of engagement, and subcategories, as well as their properties and dimensions (e.g., control and desire for autonomy), creating a cohesive picture of the data (36).

### 2.5 Rigor and trustworthiness

We relied on multiple methods to enhance the rigor and trustworthiness of the research process. One was the use of grounded theory, a well-established qualitative method with specific sampling and coding strategies. Two researchers without clinical experience collected data and performed the initial analysis, holding frequent debriefing sessions with the research team that included reflections on how their individual standpoints, identities and positions may have influenced the research process, and using an adaptation of the multiple selves reflexivity framework (39). The researcher who interviewed the service users still connected to the service was a young woman in her early 20s, a recent university graduate with a Bachelor’s degree in Psychology and working as a research assistant at the EIS clinic. The former service users and service providers were interviewed by a man in his late 20s with a Master’s degree in Education, who was a research coordinator at the EIS clinic with extensive experience in qualitative research, including grounded theory and interviewing. One concern was that service user participation may have been impacted by their awareness that researchers had some connection with the service where they received treatment. Using interviewers close in age to participating service users seemed to facilitate a friendly and open discussion. For study recruitment, service users and former service users were approached either in person or by phone regarding their participation in an interview. The interviews started with a short “warm-up” where the interviewers introduced themselves and built rapport, then explained the purpose of the study including its potential contribution to improving future EIS services. The providers were approached in person by the interviewer, who had worked with them for several years before the study. He provided them with a brief description of the study and study aims. Team members with previous clinical experience helped the interviewers by providing additional information on the general treatment experiences of service users and provider practices, allowing them to better understand the context of participant narratives. The team collectively decided on when data saturation was reached, led by the team members conducting the interviews. The study was approved by the Douglas Research Centre’s Research Ethics Board (IUSMD 11/61). All study participants provided written informed consent, and had the ability to review their transcript upon request.

## 3 Results

### 3.1 Overview of the spectrum model of engagement

**Figure 1:**
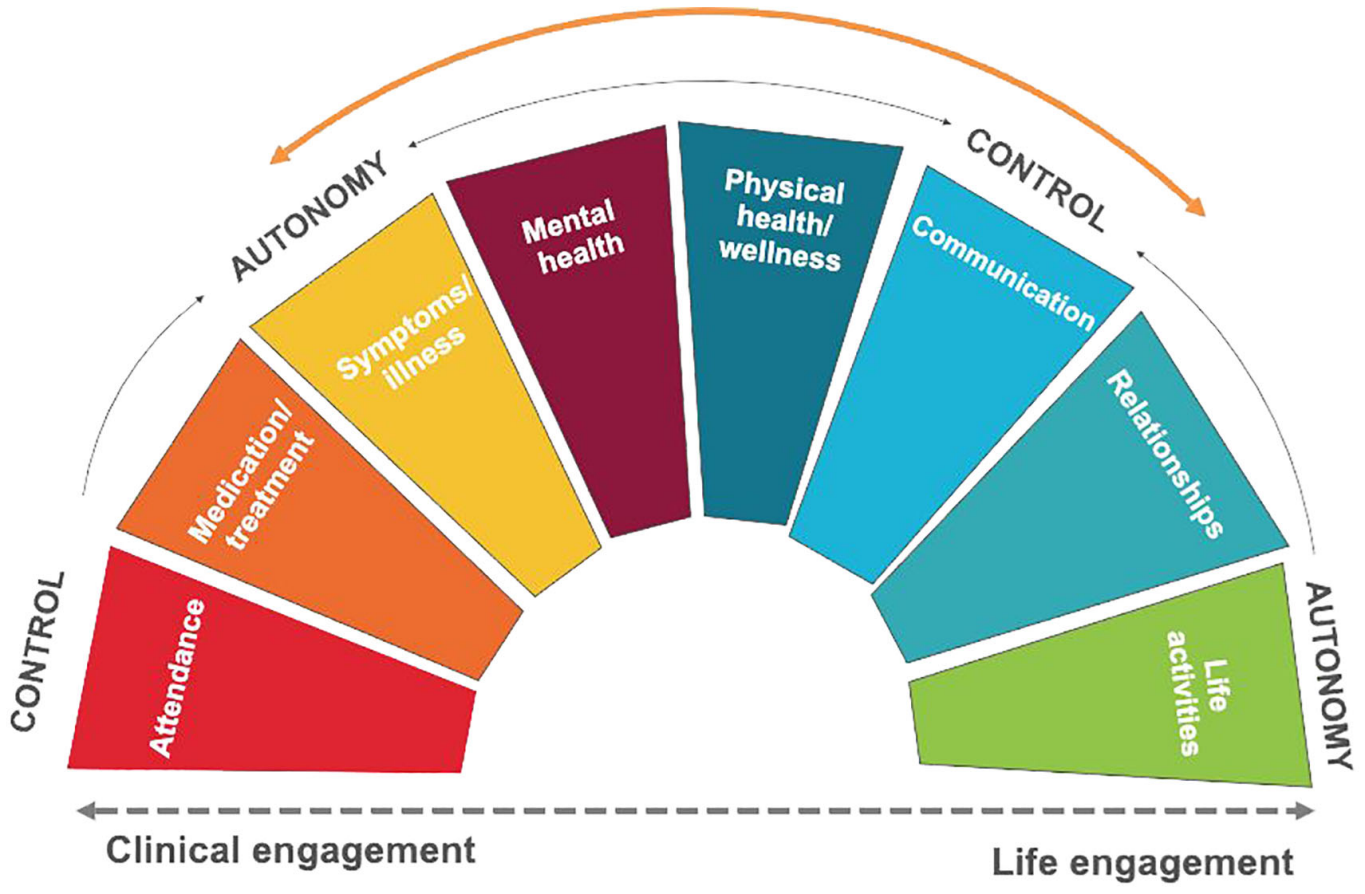
Spectrum model of engagement in services for first episode psychosis.

The study results are presented in three main sections. First, we present the eight main themes/domains of engagement as described by service users and providers: Attendance, Medication/treatment, Symptoms/illness, Mental health, Physical health/wellness, Communication, Relationships, and Life activities (**Figure 1**). The first and last themes/domains (Attendance and Life activities) describe the way in which service users and providers defined dis/engagement with EIS and, more broadly, recovery. Within these two standpoints, we position the six subdomains which relate to what appeared to be meaningful to service users and providers when receiving/offering care in EIS and during the users’ recovery journeys. Second, we identify both similarities and differences between service users and providers regarding the eight domains, unpacking the tensions between need for control and desire for autonomy in the “practice of engagement” when receiving/providing care (Section 3.3). Finally, we end the results section by reviewing the core components of the spectrum model of engagement.

### 3.2 The eight domains of engagement and differences between service users and providers

When asked during the interview, service users and providers defined dis/engagement with services and recovery in different ways. They recognized how dis/engagement can be understood as clinical engagement or attendance, but also life activities or life engagement (presented at the end of this section). Regarding Attendance, some service users attended all their appointments and followed treatment recommendations, describing a great need for support and care:

“… I feel good when I leave here. At least I know there is someone there for me…” (Service User – SU 1).

At the other extreme, some accepted help reluctantly when feeling less well and “need(ing) something extra” (SU2) or recognized a need for treatment only after “running away” and experiencing worsening symptoms (SU3). Others viewed clinic appointments as a routine “check-in” (SU4, SU5). Provider views on attendance ranged similarly from feeling responsible:

“if they don’t engage, they will return to the community, they will be disengaged and at risk for relapse … (then) return here even more sick and will take more time to recover…” (Provider – P1)

to offering services without exerting influence over user uptake.

Medication/treatment was described by service users who defined themselves as “engaged” in treatment as based on whether they were taking their medications, whereas others appealed to their autonomy in expecting to have a say with respect to taking or not taking medications. Most service providers described Medication/treatment as essential, especially in the early phases of illness, with one endorsing a proactive role for providers in setting treatment goals and encouraging medication compliance. Another service provider had a slightly different take:

“clients who … just want the medication and don’t want to deal with anything else … I don’t think they’re very engaged” (P2).

Within the domains of Symptoms/illness and Mental health, service users talked about the difficulty of using the word “psychosis” with friends and family. For some service users, the challenge of explaining psychosis and their service use clarified their own relationships to both the concept of psychosis and their experience in services. For example, one service user reported that her parents’ difficulty with psychosis as a concept made her more assertive in finding meaning for herself in this experience:

“They are very like, around the fence about psychosis and don’t really want to talk about. It’s not that way for me anymore.” (SU5)

Another service user recounted his friend’s advice not to call it “psychosis”, but a “bad trip” (SU6), leading him to alter his personal history:

“I don’t tell anybody … it’s embarrassing. I don’t want people to know I had to come to the looney bin. To be honest, when I go day-to-day, I pretend that it never happened - just put it behind me.” (SU6)

For providers, the willingness of families to support their loved ones to come to the EIS and associate with staff was a crucial factor in successful engagement. Some families discouraged treatment and viewed mental health services negatively, fearing the stigma of mental illness. Maintaining privacy or denial about having experienced/experiencing psychosis became a coping strategy for users who continued to receive treatment or visit services.

Service users gave many creative examples of how they enhanced their Physical health/wellness. Their strategies included meditation, daily routines to improve mental/physical health like walking or running, healthy diet and proper sleep, sports, going to the gym, conscious efforts to boost morale, better organization and keeping a schedule. While providers welcomed these alternative or “non-institutional” coping mechanisms, others endorsed only conventional treatment for alleviating symptoms, EIS treatment in particular, or argued that any “extracurricular” treatment needed to be evidence-based.

Regarding Communication, participants endorsed respect for service users’ voices, with some providers emphasizing their particular responsibility to express positive and hopeful attitudes. Providers who aligned more with clinical engagement took upon themselves responsibility for identifying difficulties and proposing solutions, a position welcomed by service users with the same orientation. Instead, one service user objected to doctors who “… go in there and start saying, ‘Okay, I think you should start taking this or that’” (SU7).

The reality of service user engagement grew more complex in the Relationships domain, which added the important influence and involvement of family members and friends to service user-provider partnerships in areas like attendance and treatment adherence. Some service providers saw client involvement in treating teams as highly favorable to strong therapeutic relationships, alignment of goals, and engagement. Some also recognized that successful service user engagement hinged mainly on healthy relationships and strong alliances with families, friends, or significant others, as providers navigated the delicate balance between family and service user wishes. Most service users described their families as essential supports in continuing treatment: over half of families had met with doctors or the EIS team at least once, communicated regularly with them, participated in family support groups/meetings, or accompanied them to appointments or the hospital emergency in crises. Parents made significant personal sacrifices to support service users, as illustrated below:

“My father has come like at least 20 times. Somehow, he manages to take time off, which always surprises me.” (SU8)

While some friends became closer, showing active concern, listening, and understanding, family connections were seen as key by participants. Their positive or negative reactions strongly influenced dis/engagement with services:

“I guess for the first while after I got out of PEPP, (my father) understood that I was going through something, but he gave me lots of chances. He thought that meds were a great thing (but) didn’t have a problem when I went off them.” (SU9)

Negative family relationships around psychosis developed when parents blamed service users for their condition or opposed medication, or treatment in general:

“…the pushing point was when I tried to commit suicide, which like my mum knows, but my dad doesn’t know. Which my mum had a very like, ‘that’s so selfish’ kind of attitude about, and so that’s that.” (SU3)

A few service users attended services or took medication against parental advice:

“In fact, she (mother) told me … you were born in another country, so it’s not the same culture, it’s not the same lifestyle, it happens to everybody, not just me. So, these are normal things. You have to take control, stop the medications. That’s what my mother tells me. She always tells me the same thing.” (SU14)

Similarly, friendships sometimes, but not always, faltered under the shock, disbelief, or sadness of mental illness; other friends abandoned service users and moved on, seemed at a loss for words, or were not interested:

“Well, my friends … when I got sick, they kind of walked away…. Like they didn’t know how to react, they didn’t know what to do. I have two real friends, and I talk (to them) every day…” (SU17)

“…one friend said, ‘don’t even call it a psychosis’ … and he says, ‘oh well, I’ve had bad trips, just call it a bad trip.” And I’m like, ‘that’s not even the same thing.’ A lot of my friends don’t believe in … mental health issues.” (SU6)

Life activities. As illustrated in previous sections, many service users with early psychosis were proactive in developing a comprehensive view of their recovery, not allowing mental illness to interfere with engagement in life:

“To see it as a sickness that you’re constantly trying to recover from for the rest of your life. I don’t think that’s any way to go about your life, especially if you want to strive, or you have the ability to try to do the things that you want to do.” (SU10)

Another service user privileged dimensions of life (e.g., job and living security, positive relationships, sense of agency) that supported his progress toward recovery:

“I have a job; therefore, I can feed myself, go out and do things. I have the essentials for a comfortable life and then I have the benefits of a good group of people around me that make life enjoyable. They’re motivated to move forward instead of staying where they are, which I think is better.” (SU14)

Service providers also discussed involvement in activities, like school, as a marker of engagement, or even as part of the program itself, and themselves as catalysts for reconnecting “patients” with life beyond the clinic. However, for others (service users and providers), successful engagement in life depended on staying engaged with the program.

### 3.3 Tensions and challenges in the practice of engagement

The data presented here reveal tensions between a clinical or treatment approach to engagement in EIS versus a life engagement approach that run through each domain of the spectrum model. This section further unpacks these issues and describes challenges in the practice of engagement. As shown in a series of emblematic quotations presented in **Table 2**, provider and service user responses represent both extremes of the continuum and reflect complex interactions across the eight domains. While this table provides only sample quotations from service users and providers for each domain, showing dis/alliance, similar patterns were present across all interviews.

**Table 2:** Comparison of service provider and service user perspectives favoring control and autonomy on all model dimensions.

	Attendance	Medication/ treatment	Symptoms/ illness	Mental health	Physical health/wellness	Communication	Relationships	Life activities
A. Perspectives favoring control								
Service users	“I’m engaged because I come to … appointments … take my medication. What else? I take part in whatever activity they ask me to, or any survey or anything.” (SU11)	“I came here to see the doctor. The medication he gave me was helpful. There were secondary effects, but it helped me.” (SU12)	“This is what I’ve been looking for – somebody who actually knows what they’re talking about when it comes to mental illness and prescribing things.” (SU13)	“…I have a resource … I feel comfortable because I know that when some bad things happen, I always have somebody to help me … in a way nobody else can … give me mental health advice. Because nobody knows like a doctor knows.” (SU2)	“If they give me advice, I follow it…. The advice is about my nutrition, what I eat, who I hang out with.” (SU14)	“I myself realize that it’s better to tell them what isn’t going well, and everything, and they can give you advice and help you.” (SU15)	“My whole family knows that I come here (PEPP). My mom is involved … since day one … well two years ago, because February’s going to be two years. Yeah, she comes to appointments. I like the support. My dad, he supports me but in a different way … that I’m going to school, that I’m going to keep working … to continue fighting and never give up.” (SU16)	“I get along well with my psychologist, my doctor. I never refuse anything that they try to give me; …at this point, after three years and a half, like anything that could help, I’m willing.” (SU17)
Service providers	“(engagement is) … doing a lot of accompaniment and ensuring that clients come to their appointments … participate in all the information sessions and things we give them to provide them with tools…” (P3)	“We know that compliance with medication is important in psychotic disorders, even first episode psychosis, for a year and a half, two years … I encourage the patient … tell him that this is very important. Medication is part of treatment.” (P4)	“If a patient is stable for some time, even if s/he has not finished the two years, I feel uncomfortable because I want to maintain follow-up, maybe more infrequent, just to reassure myself. If … things aren’t going so well, how worried I am depends on the symptoms … it is always necessary to think of what is best for the patient, and that really depends on the clinical situation.” (P5)	“It’s difficult because I’m trained to use the medical model to help patients … For psychosis, I don’t think … I know enough about what actually works out there aside from the medical and psychotherapeutic things … so that’s hard for me to feel comfortable with … those other methods when I don’t know if they’re going to work …” (P6)	“… we aren’t always at home or school to insure continual care. Situations of malnutrition, neglect are things we can talk about with parents. We keep an eye on (clients) to ensure that they eat well, …take care of themselves, their hygiene.” (P7)	“…just being careful with the message we’re giving and … making it very clear we’re here for you and we’re here to work on your problem and your situation…. We’re not here to tell you what to do necessarily. We’re just here to help you, coach you.” (P2)	“It’s not always easy to (balance), because I have my own perceptions of what I want for the patient …. you have to be mindful … so you don’t necessarily act on that … so that (it) becomes a primary focus of what you’re doing … But certainly, I have my ideal way …. for each patient – what I want him to do and where I want him to go…” (P6)	“Once we know how much and what medication works for this person and the symptoms start to recede…, then it’s time to kind of get the person activated in their lives to do something that will bring them … some fulfilment; some sense of … progression, that their life is moving forward.” (P3)
B. Perspectives favoring autonomy								
Service users	“I won’t come for months on end because I’m feeling so great … like I can handle everything and then sometimes I have … certain breakdowns and … need something extra … that’s where PEPP comes in …. I come when I’m not feeling so great, and then I’m good.” (SU2)	“To have a say in what my treatment options are, and if I’m going on medication, going off of it, what course we’re going on. I feel it’s important I have a say in it.” (SU5)	“My PTSD definitely doesn’t help, but otherwise I believe that if I can manage that then I’ll be perfectly fine … like my hallucinations are managed, my psychosis is managed, I’m pretty much used to it, I’ve had it since I was a kid.” (SU4)	“I find most of my treatment, …most of my improvement is my own choices. It’s what I do … I find the PEPP team helps, but what helps more are my own choices … I think the person who could help me most is myself.” (SU18)	“…being well is going to bed on time, eating well, working out, having a good state of mind …. You’ve got to take care of yourself …. Be careful with what you do … everything in moderation…. I can’t say I’m the healthiest person ever … but I try, so I’m halfway there.” (SU2)	“Maybe not talking about me in the third person to my case manager when I’m right there. Maybe not ignoring things that I say are real problems, just because they don’t understand … What else? … Give explanations to me without being patronizing…” (SU3)	“Well, my mother, she didn’t believe in taking medications. My family didn’t really believe in taking medications for depression. I don’t know what they think now that they see I’m doing better. I went online and I still researched to hear other people’s point of view by taking this medication, and that kind of like helped me… (to) have the courage to take them (pills) every day.” (SU11)	“It is true that the doctor can help me, but I have to do something to get out of this … So, it’s true that the doctor helps, but not 100%. It’s maybe 30% the doctor and 70% myself.” (SU12)
Service providers	“Their participation and collaboration in the processes of evaluation and follow-up, even if they don’t accept the recommendations or, e.g., pharmacologic treatment…. The fact that they are willing to come in, to discuss other options …” (P4)	“Engagement is not necessarily to take medication…. if the person doesn’t agree, it’s normal that what we recommend and what they do will be different. This does not mean that the person doesn’t want our help … They want changes in their life, but in a different way.” (P8)	“… some people are quite happy living with … their symptoms…. Ways of coping …. can mean … relaxation or going for walks or camping … anything that helps people feel more in tune with their selves or more comfortable with their lived experience, to me is part of the way of coping with mental health symptoms.” (P9)	“Young people can use the resources that they consider useful for themselves … Personally, I feel very much at ease that people chose other resources. Medical resources are not exclusive; they can use other resources as well…” (P5)	“If they’re engaged in certain forms of exercise; yoga, meditation … changing their nutritional habits can be of great help. Sleep hygiene is important and if they’re able to, with help or without, discover that these things are helping them … wonderful.” (P3)	“You can’t argue with them. You can insist on seeing things your way, but they have to see it too. You just say, ‘this is how I see it.’ … You make it clear that this is just my point of view, and it comes from my expertise and experience; but I am not in your shoes, and I can’t see the things that you do…” (P2)	“How they feel towards their own parents sometimes, they project that onto the psychiatrist and the case manager. Those clients are the most difficult to deal with and sometimes, in rarer cases, the family dynamic can be so toxic that the client feels that they have to choose between the family and receiving treatment. They might stop treatment for that reason; it happens.” (P3)	“When the patient decides that he has less need of the clinician, because he feels that he has attained the objectives he wanted in his life, I say to myself that this is fantastic, it’s what we want. We want them to be more engaged in their life than engaged with us.” (P8)

Regarding quotations in **Table 2A** that illustrate perspectives favoring control, providers were the party exerting control in the various domains, while service users agreed and felt comfortable with the treatment team’s directions. The quotes we selected and presented reveal that providers and service users defined engagement similarly, as attendance and active participation by the service user in clinic activities. There was also agreement between service users and service providers around compliance with medication, with providers recommending medication and service users diligently following their recommendations.

“I came here to see the doctor. The medication he gave me was helpful. There were secondary effects, but it helped me.” (SU12)

“We know that compliance with medication is important in psychotic disorders, even first episode psychosis, for a year and a half, two years … I encourage the patient … tell him that this is very important. Medication is part of treatment.” (P4)

In another example from **Table 2A** under Symptoms/illness, we quote from both a service user and a service provider who separately described service users adjusting their behavior according to symptoms, or “the clinical situation”. The provider mentioned wanting to prolong follow-up for reassurance about the service user’s wellbeing, while the service user sought greater professional expertise for the same reason. In this and the following domain, Physical health, the service providers and service users interviewed reported a strong alliance, where the provider would oversee client nutrition and self-care, and the service user welcomed this advice.

“…being well is going to bed on time, eating well, working out, having a good state of mind …. You’ve got to take care of yourself …. Be careful with what you do … everything in moderation…. I can’t say I’m the healthiest person ever … but I try, so I’m halfway there.” (SU2)

“If they’re engaged in certain forms of exercise; yoga, meditation … changing their nutritional habits can be of great help. Sleep hygiene is important and if they’re able to, with help or without, discover that these things are helping them … wonderful.” (P3)

Communication involved the provider’s ability to maintain a supportive communication style, while the service user expressed eagerness to actively inform the provider of their health status and receive advice. The dynamic under Relationships showed synergy between the service provider and service user, and with others (family members and friends) in developing meaningful relationships and a solid therapeutic alliance. However, in doing so, shared decision making was somewhat compromised when providers asserted their authority or service users followed other people’s advice or vision instead of searching for their own meanings. This last point was also well expressed in quotations on life activities showing how a provider asserted the primacy of symptom management, followed by rehabilitation, while the service user also located engagement in life within the provider’s realm of expertise.

The quotations presented in **Tables 2A, B** were organized to show that service users and service providers could find themselves in harmony, whether in favoring control or autonomy across the spectrum. However, this may not often occur. Should a service provider valuing control be working with a service user who asserts a desire for autonomy, or, by contrast, the service user is seeking guidance and support from a provider who wants the service user to focus outside of EIS, the resulting mismatch can create dissonance. As these findings reaffirmed, such potential mismatches between service providers and service users, leading to misunderstanding, lack of shared purpose, mutual distrust and ultimate disengagement existed across all domains of the spectrum.

### 3.4 The spectrum model of engagement

**Figure 1** presented the continuum from Clinical engagement (Attendance) at one end of the spectrum, to Life engagement (Life activities) at the other, and the six intermediate domains of engagement as conceptualized by service users and providers: Medication/treatment, Symptoms/illness, Mental health, Physical health/wellness, Communication and Relationships. Both service users and service providers could fall along the spectrum from autonomy to control on each of the intermediate domains. Through the process of analysis, we found intersections between various domains, for example, Symptoms/illness and Mental health; Relationships between service users and family or friends; or between these relationships and Medications, which are further explained in Section 3.2. Second, we identified both similarities and differences between service users and providers regarding the eight domains, unpacking the tensions between need for control and desire for autonomy in the “practice of engagement” when receiving/providing care (Section 3.3). The findings reveal a dynamic interplay between service user-service provider positions along the control-autonomy spectrum, and the complex array of choices involved in engaging and not engaging with services.

Participant perspectives favoring clinical engagement would tend toward stronger, more directive involvement from the providers with or without acceptance of control by service users, whereas perspectives favoring life engagement suggested greater personal autonomy of service users. The intermediate domains were independent. While a service user might prefer to give control/receive assertive direction in one domain (e.g., physical health), they might still want to assert agency in another domain (e.g., communication). Rather than a simple power balance, where autonomy is exerted by the service user and control by the provider, we conceptualized both control and autonomy as perspectives that both the service users and providers could assert. Control is therefore both “exercising control” for the provider and “asserting/giving control to the provider” for the service user, while autonomy is then both “asserted” by the service user and “supported” by the provider. Central to this framework is the philosophy that service users should always be in a position to assert and express their agency during treatment decisions and throughout their recovery – regardless of how the balance shifts between autonomy and control.

## 4 Discussion

This study presented a spectrum model of engagement in EIS for psychosis consisting of eight domains: with Clinical engagement (Attendance) and Life engagement (Life activities) at opposite ends of the spectrum, interspersed by six intermediate domains: Medication/treatment, Symptoms/illness, Mental health, Physical health/wellness, Communication, and Relationships, each bearing uniquely on engagement. According to the model, participant perspectives shifted from service provider control/service user acceptance of control to service user assertions of personal autonomy and initiative/service provider acceptance of these values, reflecting the complexity of their interactions. Many service users asserted autonomy vis-à-vis the recovery process, aiming to cope with, navigate or overcome symptoms while focusing on life activities and achievements; whereas service providers respected such aspirations, yet continued to assert their expertise and control over the recovery journey. These respective positions were not always congruent, and evolved over the recovery journey, diverging in some moments and converging in others.

The proposed spectrum model of engagement builds on and extends previous studies, providing a dynamic, interactive, understanding of dis/engagement as a fluid process centered on individual life goals (40). Engagement includes various areas of negotiation and opportunities for service users to exercise agency, whether in treatment or program structures or in their life outside the program (20, 28). Empirical studies also support the view that disengagement is not an absolute occurrence. In a longitudinal review, 56.3% of participants disengaged from EIS at least once, but 85% subsequently re-engaged, for 7.6% net disengagement (13).

The assumption that engagement and disengagement are mirror images of each other has led to simplification of a very complex process involving multiple stakeholders: service users, providers, family members and others. Engagement is a multidimensional, ongoing process. In this paper, we present a theory of service user engagement in services for psychosis through the perspectives of service users and providers, challenging many underlying assumptions about engagement in the literature, for instance, that engagement was a “status” (being engaged or being disengaged) that people needed to meet when in therapy (41), while accounting for the added complexity of tensions involving third party relationships (family, friends). Recovery advocates have questioned deeply entrenched assumptions that people with psychosis need treatment indefinitely; that service providers know best; that refusing treatment is the “wrong” choice - reflecting poor judgment or lack of insight - arguing instead that treatment choices are complex and calling for a paradigm shift from compliance to self-determination using models like shared decision-making and peer-led services (29, 42, 43). One well-known researcher and advocate, Nev Jones, has called for the inclusion of individuals with psychosis in treatment decisions, and in designing clinical programs and mental health policy (26, 44–47). Jones emphasizes the importance of empowering individuals and respecting their autonomy, valuing their preferences, goals, and agency in the decision-making process (46, 48).

Our study, along with others (40), points to an emerging understanding of engagement in EIS as a process centered on individual life goals. In another study, EIS service users described engagement with case managers as a “push-pull” process alternating between periods of good and poor engagement: while symptom exacerbation pushed them toward greater tolerance for disempowerment and engagement with services, better recovery decreased their tolerance (41). Moreover, perceived threats to personal autonomy created a barrier to professional help-seeking for mental health concerns (49). Shared decision-making and person-centered approaches are increasingly seen as key to reconciling potentially competing interests and tensions between service users and providers. In fact, in one study, service providers found that shared decision-making and patient-centered flexibility did more to facilitate engagement than assertive outreach (20).

EIS guidelines stress the importance of tailoring interventions to individual preferences, values, unique needs, experiences, and goals, going beyond symptom management and creating more personalized and meaningful treatment plans. EIS treatment plans are recommended to cover various aspects of patients’ lives: social environment, cultural background, and personal strengths. Guidelines align with recovery-oriented models (10), emphasizing empowerment, support for individual recovery journeys, and the belief that individuals can achieve their goals and lead fulfilling lives. According to EIS guidelines, the overarching goal of EIS is to provide compassionate, individualized, collaborative care to individuals experiencing early signs of psychosis (10).

This study has limitations, but also strengths. Participant interviews were based on retrospective accounts, suggesting the need for qualitative prospective designs capturing service user-provider experiences through extended treatment and recovery phases, and using multiple interview timepoints. This study was also limited to the standpoints of service users and providers, whereas, as the results show, other stakeholders like family members may play a key role in our understanding of dis/engagement and provide valuable insights around the treatment and recovery experiences of service users. Our findings suggested that family relationships tended to be positive (50), but the capacity of families to respond effectively to the crisis of psychosis depended on multiple factors (e.g., quality of parent-child relationships, family stability, their experiences with/attitudes toward illness). Previous studies have also underlined the role of caregivers in successful engagement (21, 40, 51). Although we sought feedback from service users and families (as well as service providers) in developing our interview guide, we acknowledge the lack of involvement of persons with lived experience in other fundamental aspects of the research process as an important limitation.

The strengths of our work involve methodological rigor, supported by a grounded theory approach (36). Service users recruited and interviewed included both young people attending EIS and others who had dropped out, a rare occurrence in EIS engagement studies. We also sought insights from EIS providers, a group rarely included in qualitative studies on service engagement, adding information to the equation on what Tindall called “the missing voice” (51).

## 5 Conclusions

This study sought to understand how service providers and service users make sense of, deploy, and shape (dis)engagement. Re-theorizing (dis)engagement requires a careful consideration of multiple dimensions. An examination of service user and service provider positions on the various domains identified in the spectrum model reveals a dynamic interplay between domains along the control-autonomy spectrum, as well as a complex array of choices involved in engaging or not engaging with services. In sum, as the findings described, engagement in EIS is in “harmony”, when it describes a state of balance, agreement, or compatibility between service users, providers, and other meaningful people in service users’ lives. Within this balance, service users should, and must, hold the position of “conductor” in their recovery journey, moving fluidly between asserting their desire to be guided or guiding this journey.

## Data Availability

The datasets presented in this article are not readily available because we do not believe they can be shared and maintain the confidentiality of the participants. Requests to access the datasets should be directed to Manuela Ferrari, manuela.ferrari@mcgill.ca.
